# College Life for Women Twenty Years Ago

**Published:** 1896-06

**Authors:** Mary Blair-Moody

**Affiliations:** New Haven, Conn.; Fair Haven Heights


### COLLEGE LIFE FOR WOMEN TWENTY YEARS AGO.

BY MARY BLAIR-MOODY, M. D., New Haven, Conn.

EARLY in the autumn of 1874 the majority of the faculty of the medical department of the University of Buffalo decided to favorably consider an application made some years before by a woman resident of their own city, to pursue the study of medicine in their classes on the same footing as the male students. Sex was to be left out of the question as much as possible. The minority, finding it difficult to wholly approve, yielded with true American spirit, and also welcomed the innovator with what grace they could command. My youngest brother, then about passing out of his teens, having just previously decided to pursue the study of medicine in that institution, became a member of my family, and together we went into the search for the precious possibilities of the healing art. Every day we measured our work, not by what was required, but what was possible to be done, making that also include the required. The personnel of the faculty at that time always recurs to my memory as being of a somewhat unusually high order. There was a keen love of truth among them for its own sake.

Two of them said to me at different times in the course: "Do not believe what we tell you in medicine or what anybody says until you prove it." All did not teach in that way for obvious reasons, especially the demonstrator in anatomy. It would be impossible. Prof. Hadley's classes in chemistry had been and continued always open to women. Not only so, women were cordially welcomed and encouraged to do the best work of which they were capable. Dr. Mason frequently had women visitors in his physiology classes and delighted in enlightening them on such practical points as choice of food, respiratory mechanism, effects of certain nervous lesions and similar branches of the subject.

After being well started in my work, and having become quite enthusiastic over the clearness and elegance with which the professor of surgery, Dr. Moore, instructed his classes, I chanced to be in one of the other professor's rooms when he entered without noticing me and exclaimed: "How is this that we have a woman here?" Dr. Potter entered at that moment and replied: "I assume the responsibility." He was Dean at that time, and one of them, I do not remember whom, remarked: "If we do not like it when she gets through we can close the doors." They have never been closed; but, nevertheless, it is my impression that it is liable to remain what it was in the beginning, a man's college, courteously conceding as a privilege to women advantages not taken into account by its charter members. This has its uses; chivalry on one side and gratitude on the other are encouraged. It is like a bit of the romance of the middle ages dropped into the life of a wide-awake, not to say prosaic, American city. Some of the professors said when they decided to leave the doors open to women: "It shall never be a woman's college. If they wish to study as the men do, well and good. We shall not advertise it as a college admitting women, nor make special arrangements for them, nor consult their convenience." One of the professors who took this stand and maintained it expressed at one time a thought which is shared by too many and is mentioned for that reason, "No lady will wish to study medicine," with the emphasis on lady.

To be a lady in the truest sense of the word is the worthy ambition of many American women. To be so called without possessing the qualities is scarcely a courtesy.

However it may be in other medical colleges, the last or present class is always the best in the speech of the professors. It is like the last new baby. So my class, that of 1876, took its turn in being the very best.

It was a question in my mind soon after entering how to manage, when the government is vested in the students themselves, as it is, to a great degree, in colleges of that sort. Watching for indications as to methods or ways in which to maintain my dignity as a member of the class, I soon felt that there was a majority of the students that would intelligently sustain fair play in the matter. That was a great help to me. What I would have done had that sentiment not prevailed in the class I do not know. I am sure that this sentiment also prevailed among the professors. There were a few rough fellows in the class who occasionally called my attention to themselves by cat-calls when I entered, excessive smoking at the recess, close to my seat, and other manifestations of ill-breeding. The class endured it for a while in silence, simply attempting to frown it down. It happened, however, one day that one of the ringleaders in this movement was discovered in some such trick, I do not know just what, and was doubled up and handed over the tops of the iron seats from one man to another from the back row right down to the desk and rolled over that in no gentle manner to the lecturer's usual place. The quietest, most scholarly and most gentlemenly men in the class took hold with a will and received him with open arms as he was handed along too rapidly to help himself, starting as he did doubled up like a jack-knife. This happened just before lecture time. It was done quite sternly, almost silently, and must have required no small amount of muscular force, as he was not a slight fellow to whom this dose was administered. It amused me greatly, as the man seemed to shrink from attracting attention to himself in any way afterward. It may be needless to say that he was not one of those that passed when it came to final examination. Flying overshoes was another sport that was engaged in by the students, but only occasionally did they come my way. Once when one did come I put my foot on it and there it remained through lecture time and until the owner came and asked for it.

It was my fortune to confiscate a good baseball once that came flying down my way when I was in my seat waiting for the lecture to begin. The boy who owned it playfully regained possession by virtue of superior muscular strength as we passed out of the hall after lecture. I wished them to understand that confiscation would be the rule if such things came my way, as I did not wish to engage in such recreations then and there. I think they did so understand it after the ball episode, though almost nothing was ever said on such subjects. John, the janitor, was my faithful friend through it all. He was always interested in my work, kind and helpful. Among the boys he had the reputation of being afraid of nothing, living or dead. He, too, liked to see fair play, at least so far as I was concerned, and occasionally so expressed himself to the students in his own inimitable way. "She pays her money like the rest," he said to one rough student one day. "She has just as good a right here as any one." But there were limits, by common consent apparently, to my privileges. It was to be fair play but no favor on account of sex. Our professor in medicine, magnanimous and courteous as he was to all, once overstepped the bounds, according to class sentiment. We were studying diseases of the chest under his instruction. A prize had been, or was to be, offered for the best report of a certain set of lectures on this subject. Perhaps to try the temper of the men and their opinion on the subject, he called me out at several successive clinics to make the chest examination, which was a coveted privilege and could not well be accorded to every one. About the third time a hiss of disapproval was sufficiently expressive to convey the class sentiment of fair play and no favor. A shorthand man took the prize for best report of those lectures, and also those of Dr. White on some obstetrical subject, what I have now forgotten.

One of my little lads, I gathered five about me, and one baby girl, when at home, would sometimes drive down for me at the close of our longest lecture days. It was an amusement of some of the students, or would-be students, if he came in a little before the last lecture closed, to be in the back row of seats, where he always sat, and take off his shoes and stockings. Of course, he would not be just ready when I wanted to go home. Though I was hindered a little sometimes in different ways, there was never a time when some one would not move back and give me a favorable place in the standing clinics. They could look over my head.

Dr. Hadley counseled me sometimes, sometimes gently criticised, but was understood by the class to be on the whole friendly to my work. He said to me in the beginning: "It will be easier for you to get along if you occupy yourself in lecture time by taking copious notes." Although I did not presume to compete for it, this brought me the prize in minor operative surgery, as it was given for the best reports of a long course of lectures and the shorthand men did not enter into the competition.

It was necessary to have the notes copied and in the hands of the judges on a certain Monday afternoon. As I had abandoned all thought of competing for this prize early in the term, I had not copied them. Saturday Dr. Potter called and asked me to copy them and put them in. It was then nearly noon. I immediately set about it and wrote until five o'clock Sunday morning. With as little sleep and rest as possible, the notes were not quite finished Monday noon. A clerk in my husband's employ and my brother then took hold and by four o'clock they were completed. I have since regarded this as a justifiable exception to the rule of regular sleep and rest. The temporary inconvenience which was necessary to win, was too brief to induce serious consequences. At graduation the announcement was received with rather more than the usual cheers and congratulations, six little pairs of hands in the gallery joining in the applause. As I write, the veil of years is lifted and I recall with pleasure many kindly and helpful words and acts from my teachers and associates which cannot here be written.

The greatest good to the greatest number at the smallest cost to them, was somehow inculcated along with other teaching. That principle closely followed does not tend to the accumulation of wealth.

Few, if any, of the professors remain on the faculty who were there when my class graduated in '76. Some of their places are filled by men beside whom I studied, some from the later classes, others have come from afar. The new building in closer proximity to one of the hospitals is more convenient and suitable for the numbers of women as well as men who yearly gather there for instruction. Many of the old professors have long rested from their labors, crowned with that youth which is immortal. But their precepts and examples have been good to work by. Not one of them would I have willingly exchanged for any other teacher in his department. How vividly they come to my mind day by day as I meet emergencies in practice, against which, to be forewarned, as it were, is to be forearmed. I remember well how conservatively the first of the coal products was handled by our professor of practice, who departed from his usual custom in using a remedy so purely chemical in construction and yet so complex. To me, and I believe to all, the truth was carefully imparted so far as it was known.

Once the fees were paid and the work begun there seemed to be no calculation that so much money demanded so much and no more instruction. Capacity, desire and opportunity were the only recognised limits and we were each and all encouraged to do and dare our very best.

FAIR HAVEN HEIGHTS.

